# Functional characterization of a tomato *COBRA-like* gene functioning in fruit development and ripening

**DOI:** 10.1186/1471-2229-12-211

**Published:** 2012-11-10

**Authors:** Ying Cao, Xiaofeng Tang, Jim Giovannoni, Fangming Xiao, Yongsheng Liu

**Affiliations:** 1 Ministry of Education Key Laboratory for Bio-resource and Eco-environment, College of Life Science, State Key Laboratory of Hydraulics and Mountain River Engineering, Sichuan University, Chengdu, 610064, China; 2 School of Biotechnology and food Engineering, Hefei University of Technology, Hefei, 230009, China; 3 US Department of Agriculture – Agricultural Research Service, Robert Holly Center and Boyce Thompson Institute for Plant Research, Cornell University, Ithaca, NY, 14853, USA; 4 Department of Plant, Soil and Entomological Sciences, University of Idaho, Moscow, ID, 83844-2339, USA; 5 School of Life Science and Engineering, Southwest University of Science and Technology, Mianyang, 621010, China

## Abstract

**Background:**

Extensive studies have demonstrated that the *COBRA* gene is critical for biosynthesis of cell wall constituents comprising structural tissues of roots, stalks, leaves and other vegetative organs, however, its role in fruit development and ripening remains largely unknown.

**Results:**

We identified a tomato gene (*SlCOBRA-like*) homologous to Arabidopsis *COBRA*, and determined its role in fleshy fruit biology. The *SlCOBRA-like* gene is highly expressed in vegetative organs and in early fruit development, but its expression in fruit declines dramatically during ripening stages, implying a primary role in early fruit development. Fruit-specific suppression of *SlCOBRA-like* resulted in impaired cell wall integrity and up-regulation of genes encoding proteins involved in cell wall degradation during early fruit development. In contrast, fruit-specific overexpression of *SlCOBRA-like* resulted in increased wall thickness of fruit epidermal cells, more collenchymatous cells beneath the epidermis, elevated levels of cellulose and reduced pectin solubilization in the pericarp cells of red ripe fruits. Moreover, transgenic tomato fruits overexpressing *SlCOBRA-like* exhibited desirable early development phenotypes including enhanced firmness and a prolonged shelf life.

**Conclusions:**

Our results suggest that *SlCOBRA-like* plays an important role in fruit cell wall architecture and provides a potential genetic tool for extending the shelf life of tomato and potentially additional fruits.

## Background

The ripening of fleshy fruits involves a number of physiological processes including the production of aromatic compounds, nutrients, pigmentation, and softening of flesh to an edible texture [1,2]. These processes have direct impacts not only on fruit firmness, color, flavor and nutritional content, but also on shelf life, consumer acceptability, processing qualities, in addition to pre- and postharvest disease resistance [1,2]. Excessive fruit softening is the main factor contributing to damage during shipping, storage and post-harvest handling [3]. Fruit firmness and texture also affect the integrity of chopped and diced fruit used for canning and fruit products [4]. Because postharvest losses of fresh fruits due to excessive softening can account for as much as 30~40% of total production, considerable research had focused on mechanisms of fruit softening, often using tomato (*Solanum lycopersicum*) as a model system [3].

Fruit softening during the ripening process results in part from disassembly of the cell walls, leading to a reduction in intercellular adhesion, depolymerization and solubilization of pectins, depolymerization of hemicelluloses, and loss of pectic galactose side chains [3]. Generally, the decline in fruit firmness due to softening is accompanied by elevated expression of numerous cell metabolism enzymes, including polygalacturonase (PG) [5,6], pectin methylesterase (PME) [7], β-galactosidase [8], as well as cell wall loosening proteins such as expansin [9,10]. Suppression of single genes encoding fruit PG [3,11] or PME [7,12] in transgenic tomato plants had limited impact on fruit softening during ripening, but conferred longer shelf life resulting from reduced susceptibility to postharvest pathogens. These results suggest that suppression of certain enzymes acting on cellulose, hemicellulose or pectin alone are not sufficient to prevent softening, likely due to functional redundancy of enzymes involved in what is likely a complex metabolic process [1]. Nevertheless, a recent study has shown that down-regulation of genes encoding the N-glycan processing enzymes α-mannosidase and β-D-N-acetylhexosaminidase significantly increased fruit shelf life, which was attributed to decreased softening during ripening [2]. These enzymes have been shown to break glycosidic bonds between carbohydrates, or between carbohydrates and noncarbohydrate structural molecules [13].

Expansins are cell wall-localized proteins faciliating wall loosening. They are involved in many aspects of cell wall modification during development through disruption of non-covalent bonds between matrix glycans and cellulose microfibrils [9,10,14,15]. Transgenic silencing of the tomato expansin gene *LeExp1* resulted in increased fruit firmness throughout ripening and improved fruit integrity during storage [16].

Molecular and genetic investigations have identified additional regulators of cell wall biosynthesis and regulation of cell expansion. One such activity is encoded by the *COBRA* gene previously reported in Arabidopsis, rice and maize [17-20]. The *COBRA* gene encodes a plant-specific glycosylphosphatidylinositol (GPI)-anchored protein with a ω-attachment site at the C terminus, a hydrophilic central region, a CCVS domain, a potential N-glycosylation site, an N-terminal secretion signal sequence, and a predicted cellulose binding site [21]. It has been reported that COBRA localizes at the external plasma membrane leaflet through a glycosylphosphatidylinositol (GPI) moiety [22]. Genetic impairment of the *COBRA* gene results in reduced levels and improper orientation of crystalline cellulose microfibrils in Arabidopsis and rice [17,18,22,23]. Despite the many studies of the *COBRA* gene in several plant species, little has been learned concerning *COBRA* ortholog(s) in tomatoes, the model system for fleshy fruit development and ripening. Here we report functional characterization of a tomato *COBRA* gene (*SlCOBRA-like*). We specifically demonstrate its role in early fruit development and the potential for enhanced fruit firmness and shelf life by manipulating its expression in maturing transgenic tomato fruits.

## Results

### COBRA gene family members in tomato

In order to identify tomato orthologs of the *COBRA* gene, we ran a BLAST search in the SOL Genomics Network (SGN, http://solgenomics.net/) using the Arabidopsis *COBRA* gene sequence (*AtCOB*, accession No. AF319663.1) as query. 17 unigene tomato cDNAs homologous to *AtCOB* were found in the SGN database and were designated as *SlCOBLs* (Table 1). All of the corresponding predicted amino acid sequences contain the characteristic Interpro IPR006918 or IPR017391 omain of the plant *COBRA-like* gene family. These *Sl*COBL members, together with other plant COBRA-like proteins from Arabidopsis, rice and maize, can be grouped in two clades of a phylogenetic tree. 11 *Sl*COBL members consisting of 5~7 exons were clustered into clade I and the other 6 members (containing 2 or 3 exons) were grouped in clade II (Additional file 1: Figure S1). We focused further analyses on *SlCOBRA-like* as it appeared to be constitutively expressed in many tomato tissues suggesting an essential role in tomato biology. A total of 99 *SlCOBRA-like* ESTs were found in the SGN EST database, which were derived from various tissues including leaf, flower and developing fruits.

**Table 1:** **Tomato *COBRA *gene family in The SOL Genomics Network (SGN) and their sequence characteristics**

**Family name**	**Gene code (SGN)**	**Exons**^**a**^	**Amino Acids**^**b**^	**CCVS domain**	**N-terminal signal peptide**	**Potential ω- site**
SlCOBL4	Solyc01g065530.2.1	2	648	yes	yes	S(624)
SlCOBL5	Solyc01g103860.2.1	5	423	yes	no	no
SlCOBL6	Solyc01g111050.2.1	5	469	yes	no	no
SlCOBRA-like	Solyc02g065770.2.1	6	444	yes	yes	N (417)
SlCOBL7	Solyc02g080240.1.1	2	385	no	yes	no
SlCOBL8	Solyc02g080250.1.1	3	342	yes	no	no
SlCOBL1	Solyc02g089120.2.1	6	447	yes	yes	N(420)
SlCOBL9	Solyc02g089130.2.1	6	434	yes	yes	N(410)
SlCOBL10	Solyc03g070440.2.1	7	431	yes	yes	no
SlCOBL11	Solyc03g070460.1.1	3	116	no	no	no
SlCOBL12	Solyc03g114880.1.1	7	319	no	no	no
SlCOBL2	Solyc03g114890.2.1	6	401	yes	yes	no
SlCOBL13	Solyc03g114900.2.1	6	447	yes	yes	no
SlCOBL14	Solyc03g114910.2.1	6	458	yes	yes	N(430)
SlCOBL3	Solyc07g064200.2.1	3	615	yes	yes	no
SlCOBL15	Solyc09g075540.1.1	2	652	yes	yes	no
SlCOBL16	Solyc10g006450.2.1	2	671	yes	yes	no

^a^ The number of Exons. ^b^Amino acid residue number in each predicted SlCOBL protein. Predictions of N-terminal signal peptide and potential C-terminal GPI-Modification sites (ω- sites) were performed with SignalP Version 3.0 ( http://www.cbs.dtu.dk/services/SignalP/)[49] and big-PI ( http://mendel.imp.ac.at/gpi/gpi_server.html) [50], respectively.

### Expression of *SlCOBRA-like* in tomato

The full-length *SlCOBRA-like* cDNA was isolated from tomato seedlings by RT-PCR using gene-specific primers (Additional file 2: Table S1). The deduced *Sl*COBRA-like protein contains a central cysteine-rich domain (CCVS domain), a N-terminal secretion signal sequence for targeting to the endoplasmic reticulum, a highly hydrophobic C terminus and the ω-site required for processing the C-terminal [21] (Table 1). Moreover, several potential N-glycosylation sites frequently associated with GPI-anchored proteins and extracellular proteins, as well as one HMM-predicted putative cellulose binding domain II (E value =0.018) were observed in the *Sl*COBRA-like sequence (Additional file 3: Figure S2) [17,21,22]. Basic Local Alignment Search Tool (BLAST) analysis showed that *Sl*COBRA-like shares 63~80% similarity with other COBRA proteins from Arabidopsis, *Oryza sativa*, and *Zea mays*[17,18,20,24]. Phylogenetic analysis revealed *Sl*COBRA-like shares the highest amino acid identity with *At*COBL1, thus localizing within in the same clade (Additional file 1: Figure S1).

**Figure 1:**
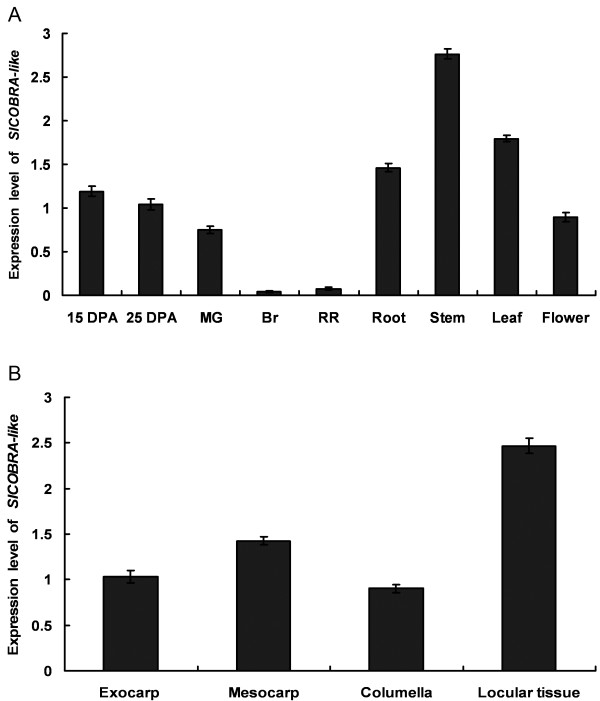
**Expression pattern of the *SlCOBRA-like *gene in tomato. (A)** Expression of *SlCOBRA-like *in various tissues. Total RNAs were extracted from seedling roots, seedling stems, fully expanded leaves, flowers and pericarp at different developmental stages (15 day-post anthesis, DPA; 25 DPA; MG, mature green, 35 DPA; Br, Breaker and RR, 7 days after Breaker, respectively).** (B)** Spatial expression of *SlCOBRA-like * in fruits at 15 DPA. Real-time RT-PCR analysis was performed as described in Methods. Average values and standard errors are shown from three independent replicates.

We also conducted real time RT-PCR analysis and found that the *SlCOBRA-like* gene is predominantly expressed in roots, stems, leaves, flowers and early development fruits. However, the expression level in fruits declined dramatically at the breaker and later ripening stages (Figure 1A), implying a possible role in early fruit development. In addition, we isolated total RNAs from exocarp, mesocarp, columella or locular fruit tissue from immature fruit 15 days post anthesis (DPA) for RT-PCR analysis. The results showed that the *SlCOBRA-like* expression level is relatively high in locular tissue as compared to other tissues at this specific developmental stage (Figure 1B).

### Transgenic tomatoes whose endogenous *SlCOBRA-like* gene was repressed displayed abnormal fruit phenotypes

**Figure 2:**
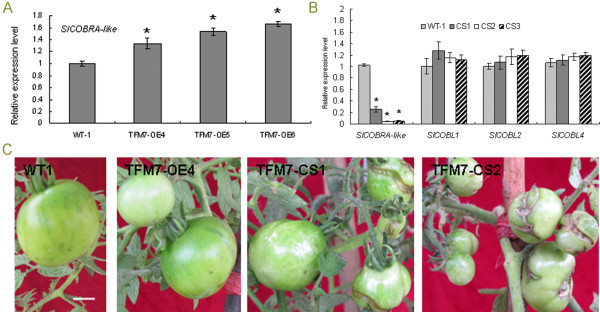
**Altered expression of *SlCOBRA-lik*e and cracking phenotype in transgenic tomato fruits. (A) ***SlCOBRA-like *mRNA levels in immature fruits (15 DPA) of three independent overexpression (OE) lines. **(B)** Expressions of *SlCOBRA-like* and three *SlCOBL* genes in immature fruits (15 DPA) of three independent co-suppression (CS) lines. mRNA levels were analyzed by real-time RT-PCR. Each bar represents three repetitions from each RNA sample (derived from pools of three fruits per plant). Significance was calculated using the Student’s *t* test. Asterisks indicate statistical differences as compared to WT1 (P <0.005*). **(C)** Representative immature fruits of wild-type WT1, transgenic lines TFM7-CS1, TFM7-CS2 and TFM7-OE4. Bar =1cm.

To determine the role of *SlCOBRA-like* in fruit development, transgenic tomato plants over-expressing the *SlCOBRA-like* cDNA in fruits were generated. The *SlCOBRA-like* transgene was driven by a fruit-specific *TFM7* promoter [25]. Twenty independent transgenic lines were generated. As expected, several transgenic lines (including TFM7-OE4, TFM7-OE5 and TFM7-OE6) overexpressing *SlCOBRA-like* (OE) were confirmed by RT-PCR analyses (Figure 2A). None of these OE lines exhibited altered fruit phenotypes (Figure 2C). However, three of the twenty transgenic lines exhibited extensive non-uniform cracking on the surface of their immature green fruits (Figure 2C). RT-PCR analyses revealed that in cracked transgenic fruits (lines TFM7-CS1, TFM7-CS2, and TFM-CS3), the endogenous *SlCOBRA-like* expression was substantially co-suppressed (CS) as compared to non-transformed wild type (WT) control plants (Figure 2B). We then generated transgenic tomato plants with RNA interference (RNAi)-based repression of the *SlCOBRA-like* gene to confirm this repression effect. Unfortunately, none of our RNAi transgenic lines displayed significant repression of the *SlCOBRA-like* gene (data not shown). One possibility is that more complete suppression of the *SlCOBRA-like* gene by RNAi in culture tissues than resulting from CS may have caused lethality in early development.

Because there are 17 *COBRA* members in tomato, it was necessary to verify the specificity of suppression of *SlCOBRA-like* in the 3 available CS lines. We examined the expression of *SlCOBL1*, *2* and *4* in CS fruits. There were two reasons we selected these 3 *SlCOBLs*: firstly they represent high (86.6% identical), medium (59.9% identical), and low (17.9% identical) similarity to *SlCOBRA-like*, respectively; secondly the fruit-derived ESTs of these 3 *SlCOBLs* were found in the SGN EST database, suggesting they are expressed in fruits, the tissues where phenotypes were most apparent. As shown in Figure 2B, the real-time RT-PCR assay indicated the expression of *SlCOBL1*, *2* and *4* was not affected in CS lines, suggesting that fruit cracking was caused specifically by repression of *SlCOBRA-like* expression.

### Anatomical alterations of fruit pericarps in transgenic plants with altered *SlCOBRA-like* expression

**Figure 3:**
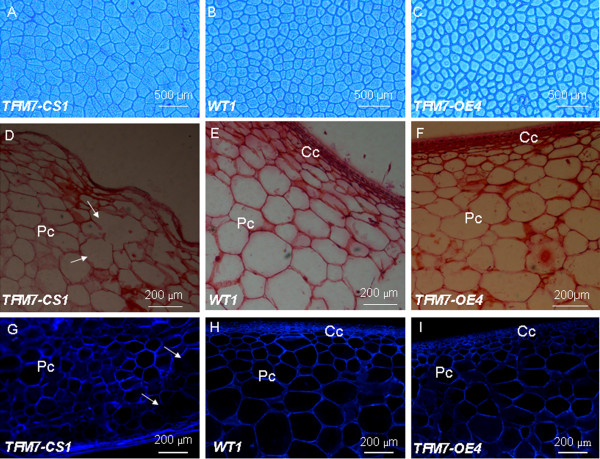
**Microscopic analyses of fruit epidermal cells and pericarp sections during early fruit development. (A-C)** Epidermal cells at the surface of immature green fruits (15 DPA). Photographs were taken with a Leica LDM 2500 microscope using a blue filter. **(D-F)** Paraffin transverse sections of pericarp tissues from MG fruits stained with Safranin O (a cell wall-specific dye). **(G-I)** Transverse sections of pericarp from MG fruits stained with Calcofluor White and visualized under UV light with a fluorescence microscope. Pc, parenchymatous cell; Cc, collenchymatous cell. The collapsed walls in parenchymatous cells of mesocarp tissues (**D** and **G**) are indicated by arrows. Sections were isolated from the same location in all fruits.

Since the exocarp (epidermis) plays important roles in determining the rate of expansion and mechanical support for the entire fruit [26], light microscopy analysis was conducted to examine possible changes in this tissue of transgenic fruits. 15 DPA fruit exocarp sections were mounted in 15% HCl and photographed using a Leica LDM 2500 microscope. As shown in Figures 3A-C, the epidermal cell layer of fruit exocarp displayed no significant differences (the Student’s *t* test, p=0.48) in epidermal cell size (not including cell wall) between WT (26.25±1.45 10^-3^ mm^2^) and TFM7-OE (27.04 ± 0.62 10^-3^ mm^2^). However, the epidermal cell size of TFM7-CS exocarp tissue was 45.52 ± 3.01 10^-3^ mm^2^, far exceeding that of the WT or TFM7-OE. In addition, the epidermal cells of TFM7-CS pericarp expanded radially to a far greater extent than those of the WT or TFM7-OE lines. In contrast, the epidermal cell separation (spacing between neighboring epidermal cells) was significantly altered in both TFM7-OE (29.11±2.69 μm) and TFM7-CS (11.82±3.84 μm) fruits, as compared with that in WT (18.43± 1.75 μm), suggesting thicker epidermal cell walls in TFM7-OE fruits but thinner epidermal cell walls in the TFM7-CS fruits, respectively.

To further compare the pericarp structure of fruits in WT and transgenic lines, detailed cytological analysis was performed. Anatomical paraffin sectioning and Calcofluor staining of pericarp tissue (MG stage, 35 DPA) revealed an increased number of collenchymatous cells beneath the epidermis in the TFM7-OE fruits compared to WT (Figure 3E, F, H, and I). In contrast, the TFM7-CS fruits displayed a "waviness" of the surface with an apparent lack of cuticle and an abnormal shape and size-distribution of the cells, particularly in the thin layer of elongated epidermal cells at the fruit surface. The small cells beneath the epidermis were almost absent in the TFM7-CS fruits and some collapsed parenchymatous cells in the mesocarp were observed in TFM7-CS (Figure 3D and G). Taken together, these results suggest that the *SlCOBRA-like* gene plays important roles in pericarp cell wall development of immature fruit.

### Analysis of cell wall macromolecules by Fourier Transform Infrared (FTIR) spectroscopy

**Figure 4:**
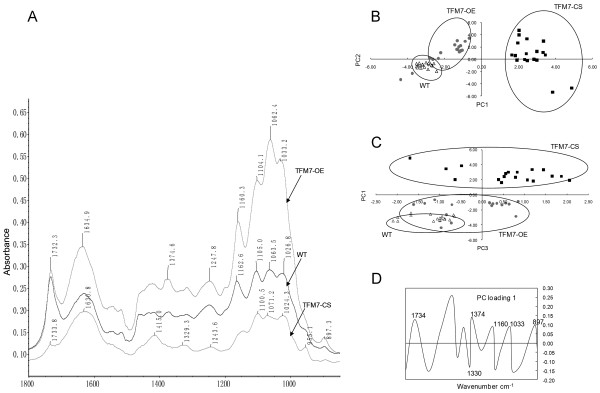
**FTIR analyses of pericarp cell walls from fruits at the RR stage. (A)** Representative FTIR spectra of pericarp cell walls from WT1 and transgenic lines (TFM7-OE4 and TFM7-CS1).** (B** and **C)** PCA scores plot in principal component analysis (PCA) of FTIR spectra. Scores obtained on PC1-PC2 **(B)** or PC1-PC3 **(C)** clearly discriminated transgenic TFM7-CS spectra from TFM7-OE and WT spectra. **(D)** PC1 loading for analyzed characteristic peaks in FTIR spectra. Exploratory PCA was performed using the complete set of 54 FTIR spectra (18 FTIR spectra for each population).

To further investigate the effect of altered *SlCOBRA-like* expression on cell wall macromolecules, a rapid assay for macromolecule composition in the WT and transgenic pericarp cell walls was performed using FTIR spectroscopy. As shown in Figure 4A, the characteristic peaks of functional cellulose groups (wave numbers 1374, 1164–1160, 1102, 1063–1060 and 1033–1026 cm^-1^) [27,28] in the spectra were higher in the TFM7-OE fruits, but lower in the TFM7-CS fruits, as compared to WT. These results indicate that, compared with the WT, TFM7-OE cell walls contain more crystalline cellulose, whereas TFM7-CS has less.

In addition, exploratory principal component analysis (PCA), a statistical method usually used for discriminant analysis of spectroscopic data [29], was performed for the complete set of 54 FTIR spectra collected from the WT and transgenic pericarp tissues (18 FTIR spectra for each population). Three principal component loadings (PCs) were extracted, among which PC1 accounted for 61.74% of the total variability, and with characteristic cellulose peaks (Figure 4D). The PCA scores of PC1-PC2 or PC1-PC3 revealing the difference between the WT and transgenic pericarp tissues were pronounced (Figure 4B and C): in this scatter plot, the cluster of TFM7-CS samples showed a clear separation from clusters of the WT or TFM7-OE samples, despite the fact that only subtle differences between the WT and TFM7-OE samples were observed, and in which slightly more positive scores were detected in TFM7-OE samples and more negative scores in WT samples (Figure 4B).

### Altered cell wall composition of transgenic fruits with altered *SlCOBRA-like* expression at the red ripe (RR) stage

**Figure 5:**
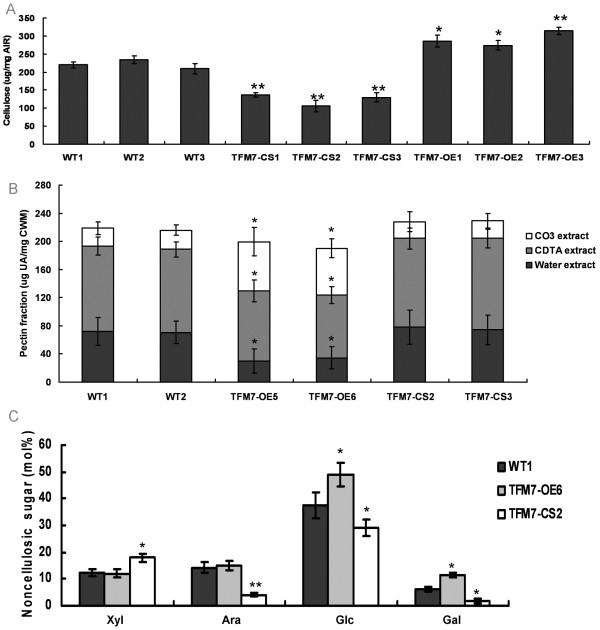
**Pericarp cell wall components of WT and transgenic fruits at the RR stage. (A)** Cellulose content (micrograms per milligram alcohol-insoluble residue [AIR]) in the cell walls of pericarp tissues. **(B)** Uronic acid (UA) content in sequentially extracted pectin fractions of the pericarp cell wall materials (CWMs). **(C)** Noncellulosic neutral sugar content in the trifluoroacetic acid (TFA)-soluble wall fractions (mole percent of total wall neutral sugars). The error bars represent three measurements from each AIR or CWM sample. Significance was calculated using the Student’s *t* test. Asterisks indicate statistically significant differences (P <0.005** or P <0.05*).

We next investigated the impact of altered *SlCOBRA-like* expression on fruit cell wall composition in transgenic fruits. Cellulose quantification analysis of the pericarp cell wall [30] was performed on WT and transgenic RR fruits. Cellulose levels were significantly higher (average 1.46-fold) in TFM7-OE pericarp than in WT, but they decreased about 40% in TFM7-CS pericarps (Figure 5A). Since pectin modification is important for textural changes during fruit ripening, pectin fractions were extracted using a sequential series of solvents [31] and measured as the amount of galacturonic acid (UA). As shown in Figure 5B, the amount of covalently bound pectin (Na_2_CO_3_ extract) in TFM7-OE fruits was higher than that in WT or TFM7-CS fruits. Moreover, the soluble cell wall fraction was significantly lower in the TFM7-OE fruits than in WT or TFM7-CS (Figure 5B). The ratio of bound pectin (CDTA+CO_3_ extracts) to soluble pectin was significantly higher in TFM7-OE fruits (4.5~5.0) than in WT (2.0) or TFM7-CS (1.9~2.0).

To quantify sugar composition in fruit cell walls, the trifluoroacetic acid (TFA)-soluble (noncellulosic) wall fraction was converted to alditol acetates and analyzed by gas chromatography (GC) [32]. As shown in Figure 5C, the TFM7-OE fruit cell wall was found to possess more noncellulosic glucose (Glc) and cell wall galactosyl (Gal) residues. In contrast, the TFM7-CS fruit cell wall had less arabinose (Ara), Glc or Gal, but more xylose (Xyl).

### Altered fruit texture and shelf life in transgenic fruits

We conducted penetration and compression analyses (TA-XT Plus, Stable Microsystems) to determine the texture parameters of WT, TFM7-OE or TFM7-CS fruits at different developmental stages. For all genotypes the skin mechanical strength and fruit firmness, represented by penetration mass and compression mass respectively, dramatically decreased from MG to RR, while fruit skin elasticity (represented by penetration distance) did not significantly change between the MG and RR stages (Table 2). However, skin puncture strength was greater in TFM7-OE fruits, but lower in TFM7-CS, than in WT, and enhanced skin elasticity was also observed in TFM7-OE fruits. In addition, despite a small reduction in firmness at the MG stage, TFM7-OE fruits were 1.6 to 1.9-fold firmer than WT at the RR stage. In contrast, the compression mass of TFM7-CS fruits was about 33% that of WT fruits at the same stage (Table 2). Thus, these results suggest *SlCOBRA-like* gene activity manifests in fruit texture phenotypes.

**Table 2:** Textural analyses of fresh intact fruits from wild-type (WT) and transgenic lines

	**Sample**	**Penetration mass**^**a**^**(g) ±SE**	**Penetration distance**^**a**^**(mm)±SE**	**Compression mass**^**b**^**(kg)±SE**
Mature green	WT-1	66.14±7.10	0.31±0.05	13.23±0.45
	TFM7-OE-4	83.90±5.21*	0.59±0.02**	13.15±0.69
	TFM7-OE-5	82.42±9.01*	0.51±0.03**	14.43±0.21*
	TFM7-OE-6	93.84±9.75*	0.65±0.04**	16.08±1.05*
	TFM7-CS-1	40.62±6.23*	0.29±0.07	10.1±0.75**
	TFM7-CS-2	41.09±5.67*	0.32±0.04	9.89±0.87**
	TFM7-CS-3	44.32±8.21*	0.27±0.06	9.14±0.93**
Red ripe	WT-1	50.45±3.05	0.35±0.05	2.38±0.32
	TFM7-OE-4	68.08±4.21**	0.61±0.07*	3.97±0.22**
	TFM7-OE-5	64.13±5.65*	0.60±0.05*	4.02±0.54**
	TFM7-OE-6	65.41±6.93**	0.67±0.09*	4.56±0.26**
	TFM7-CS-1	27.94±5.91**	0.33±0.07	1.61±0.28*
	TFM7-CS-2	29.57±4.66**	0.31±0.05	1.59±0.34*
	TFM7-CS-3	31.81±2.33**	0.39±0.09	2.03±0.59

Texture of fresh intact fruits for wild-type (WT-1) and three independent transgenic overexpressing (TFM7-OE4, 5 and 6) and cosuppressing (TFM7-CS1, 2 and 3) lines was analyzed using TA-XT Plus (Stable Microsystems).

^a^ indicated that penetration analysis of fresh fruits with a 2mm Cylinder Probe (P2/N); Three measurements for each fruit (6 fruit per individual plant) were taken at each stage. Value was showed as mean ±SE (n=18).

^b^ indicated that compression analysis with a 100 mm compression platen (P/100). Value was showed as mean ±SE (n=15).

The significance was calculated by the student’s *t* test. Asterisks indicated statistical differences to WT (P <0.005** or P <0.05*).

**Figure 6:**
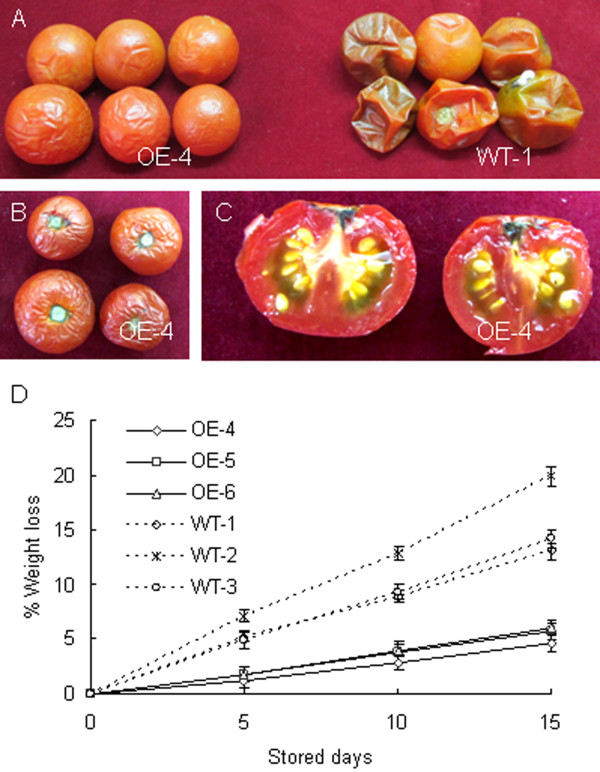
**Overexpression of *SlCOBRA-like *prolongs fruit shelf life. (A)** RR fruits from TFM7-OE4 (left group) and WT (right group) plants after storage at room temperature (23~25°C in 55~60% relative humidity) for 40 days. **(B)** and **(C)** The opposite side and longitudinal section of TFM7-OE4 fruits after 40 days of storage, respectively. **(D)** Relative fresh weight loss in fruits. The weight loss per fruit was calculated every five days until they lost their texture and structural integrity. Values represent means ±SE (n=6).

The enhanced mechanical strength and fruit firmness of TFM7-OE prompted us to assess whether overexpression of *SlCOBRA-like* can extend fruit shelf life. WT and TFM7-OE fruits were harvested at the RR stage (7 days after Breaker) and stored at the same condition until they reached complete deterioration. We found that during storage, TFM7-OE fruits lost less weight (water) than WT fruits (Figure 6D). In fact, WT fruits started to shrink 15 days after storage, with effusion of juice contents and loss of texture and integrity, whereas the TFM7-OE fruits did not display such signs of deterioration until 40 days later (Figure 6A, B, and C).

### Suppression of *SlCOBRA-like* results in up-regulation of cell wall-degradation and cell wall-based signalling genes

**Figure 7:**
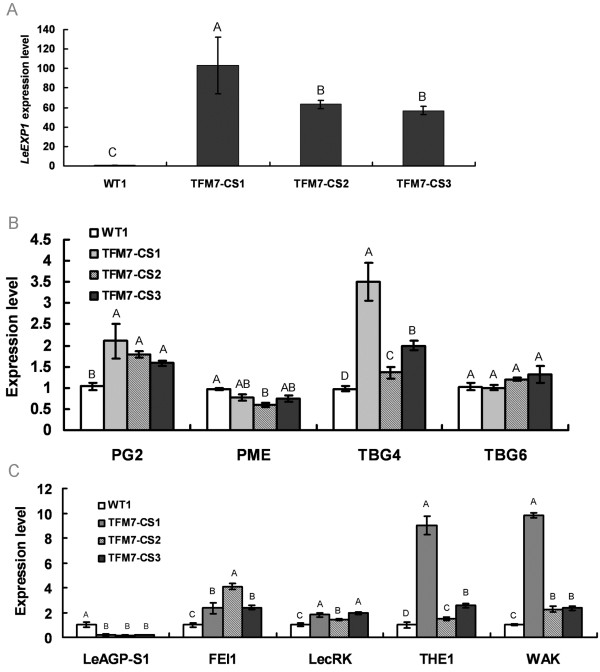
**The impact of altered *SlCOBRA-like *expression on genes involved in cell wall degradation and cell wall-based signalling.** Real-time RT-PCR analyses revealed up-regulation of genes encoding enzymes involved in cell wall metabolism **(A** and **B)** and additional genes encoding cell receptor-like kinases **(C)** in immature green fruits (15 DPA) from three independent CS lines. Each bar represents three repetitions from each RNA sample (derived from pools of three fruits per plant). Significance was determined by LSD (least significant difference) multiple comparison. Letters above each bar represent groupings of statistical significance (P<0.01).

The morphological alterations in TFM7-CS transgenic fruits prompted us to investigate whether *SlCOBRA-like* impacts genes encoding proteins involved in cell wall-degradation. Since the *SlCOBRA-like* mRNA level is normally low at ripening, immature CS transgenic fruits at 15 DPA were selected for analysis. As shown in Figure 7, elevated mRNA accumulation of *polygalacturonase* (*PG*), *β-galactosidase* (*TBG4*) and *expansin* (*LeExp1*) was detected in TFM7-CS fruits. Notably, *LeExp1* transcripts increased 50~100 fold. In addition, several genes encoding receptor-like kinases (RLKs) were also up-regulated in TFM7-CS fruits, including wall-associated kinase (WAK), THESEUS1 (THE1), FEI and lectin receptor kinase (LecRK) (Figure 7C), suggesting that some of the relevant signalling pathways in the extracellular matrix that control the structure and integrity of cell wall were influecned by loss of *SlCOBRA-like* expression homeostasis. It is notable that there was considerable variation of the induction levels of these genes among the 3 TFM7-CS fruits. We speculate this could be due to the different degrees of suppression of *SlCOBRA-like* in the 3 TFM7-CS transgenic fruits (Figure 2B).

## Discussion

*COBRA* belongs to a multigene family consisting of 12 members in Arabidopsis, 11 in rice, and 9 in maize [18,21,33]. Arabidopsis *COBRA*, *AtCOBL4*, maize *ZmBK2* as well as rice *OsBC1* have been shown to be required for cellulose synthesis [17-20,24], while Arabidopsis *AtCOBL9* and maize *ZmBk2L1* impact tip-directed growth during root hair development [34-37]. The newly released tomato genome sequence suggests there are at least 17 *COBRA* family members in tomato (Table 1). However, the role of *COBRA* in fruit development and texture has until now remained elusive. Our study addresses this question and provides a potential strategy for manipulation of fruit firmness and shelf life of tomatoes via modification of *SlCOBRA-like* expression.

### The role of the *SlCOBRA-like* gene in fruit development

The *SlCOBRA-like* gene was highly expressed during the early stages of fruit development, but the expression level dramatically declined after the breaker stage (Figure 1A), indicating a role in early fruit development. This was further supported by phenotypic analysis of fruit–specific suppression of the *SlCOBRA-like* gene in the TFM7-CS transgenic tomato plants, which exhibited anatomical changes of fruits during early development. It is worth noting that there are 17 *COBRA* members in tomato, therefore it was necessary to verify the specificity of suppression of *SlCOBRA-like* in the 3 TFM7-CS lines to allow accurate interpretation of our results. RT-PCR showed that 3 *SlCOBLs*, representing the highest, medium and low similarity to *Sl*COBRA-like, were not repressed in the TFM7-CS fruits (Figure 2B), suggesting suppression of *SlCOBRA-like* is likely to be specific. However, it is possible that other *SlCOBL* members also play important roles in fruit development. Further characterization of other *SlCOBL* members, particularly *SlCOBL1/4/9/14* that also contain all characteristic domains of COBRA (Table 1) and are expressed in fruit, will help to address this question.

### Effect of the *SlCOBRA-like* gene on fruit cell wall biosynthesis and integrity

Overexpression of the *SlCOBRA-like* gene in transgenic fruits resulted in a significant increase of cellulose content (Figure 4A and 5A). On the other hand, more cell wall-bound Na_2_CO_3_-soluble pectin and cell wall galactosyl residues were found in the TFM7-OE RR fruit cell wall (Figure 5B, C), indicating that overexpression of *SlCOBRA-like* is responsible for less cell wall macromolecule solubilization/depolymerization during fruit ripening. These results, together with the cracking phenotype of the TFM7-CS immature fruits, suggest that the *Sl*COBRA-like protein is involved in not only regulating cellulose synthesis but also maintaining integrity of cell walls during processes of extension and assembly. Previous studies have also demonstrated the complexity of cell wall integrity. For example, the *brittle* phenotype, observed in rice *bc1*[18], Arabidopsis *cobl4*[19] and maize *bk2*[24], is not necessarily a result of cellulose deficiency because Arabidopsis *cellulose synthases* (*cesAs*) mutants with a reduction in cellulose content did not display the *brittle* phenotype [38].

*SlCOBRA-like* may also play an important role in fine-tuning the expression of several genes encoding enzymes involved in cell wall degradation and cell wall-based signalling. *PG*, *TBG4* and *LeExp1* mRNAs were elevated in immature TFM7-CS fruits (Figure 7A, B). These genes encode cell wall-degrading proteins and are normally induced at ripening (BR) and throughout later ripening [8,9,39]. In contrast, little if any change in expression was detected in *PME* and *TBG6* (Figure 7B), whose expression usually declines rapidly as fruit begin to ripen [8,40,41]. It is also notable that the down-regulation of *SlCOBRA-like* led to elevated expression of genes encoding several receptor-like kinases (RLKs), which can relay a signal to the cytoplasm via the cytoplasmic kinase domain (Figure 7C) [42,43]. Similar to COBRA proteins, arabinogalactan-proteins (AGPs) usually have an N-terminal GPI anchor site [44] and play important roles in cell expansion, proliferation and differentiation [44,45], and signal transmission between the cell wall and cytoplasm [46]. Interestingly, repression of *SlCOBRA-like* in TFM7-CS immature fruits repressed the mRNA accumulation of tomato *LeAGP-S1* encoding the S1 subunit of AGP (Figure 7C), thus suggesting genetic interaction between *COBRA* and *AGP* in fruit cell wall development. However, the mechanism underlying this interaction remains to be elucidated.

#### Overexpression of *SlCOBRA-like* enhances fruit firmness and shelf life

The plant cell wall is a highly organized fibrillar network providing mechanical support for cells, tissues, organs and the entire plant body [43]. It was suggested that over 400 annotated proteins are localized in the cell wall (Arabidopsis Genome Initiative [AGI], 2000) and more than 1,000 genes in the genome are implicated in cell wall biogenesis and modification [47]. Moreover, cell wall modifications have been implicated to be the major determinant of fruit softening, although changes in turgor pressure, anatomical characteristics, and cell wall integrity are also likely to play significant roles [11]. In fact, transgenic manipulation of the activities of single cell wall-modifying enzymes in transgenic tomatoes had little impact on fruit softening during ripening [3]. Here we show that although *SlCOBRA-like* is primarily expressed in early fruit development, it is required for normal fruit softening during ripening specifically through its reduced repression. Enhanced expression of *SlCOBRA-like* in transgenic TFM7-OE fruits conferred increased fruit firmness and extended postharvest shelf life (Table 2; Figure 6). Increased firmness might be due to both an increase in cellulose content also in addition to changes in pericarp anatomical structure, especially in the form of increased numbers of sub-epidermal collenchymatous cells. It has been shown that changes in pericarp architecture can have a profound impact on fruit firmness. Guillon and co-workers reported that suppression of the tomato *DR12* gene (an auxin response factor) caused unusual pericarp cell division and a higher proportion of sub-epidermal collenchymatous cells, resulting in pleiotropic phenotypes including enhanced fruit firmness similar to what we report here for *SlCOBRA-like* overexpression [48].

## Conclusions

We present data demonstrating that the *SlCOBRA-like* gene plays an important role in regulation of cell wall architecture during fleshy fruit development. Transgenic plants overexpressing *SlCOBRA-like* exhibited enhanced fruit firmness and prolonged shelf life. While aspects of regulation of *SlCOBRA-like* expression and cell wall modification in tomato fruit development remain open to further investigation, our study provides a potential strategy for genetic manipulation of improved fleshy fruit quality and shelf-life via altered *COBRA* expression.

## Methods

### Plant material

Tomato plants (*Lycopersicon esculentum* cv. *Alisa Craig*) were grown in a greenhouse under natural light and irrigated manually every other day. For cytological, texture, cell wall composition and molecular analysis, fruits of WT and T_1_ generation transgenic lines were harvested at the immature green (15 DPA), mature green (MG, 35 DPA), Breaker (BR), and Red Ripe (RR, 7 days after BR) stages after tagging of flowers at anthesis.

### Amino acid sequence analyses

Signal peptide and GPI modifications were predicted with SignalP Version 3.0 (http://www.cbs.dtu.dk/services/SignalP/) [49] and big-PI (http://mendel.imp.ac.at/gpi/gpi_server.html) [50], respectively. N-glycosylation site prediction was performed using NetNGlyc 1.0 (http://www.cbs.dtu.dk/services/NetNGlyc/). SUPER-FAMILY 1.69 (http://supfam.mrc-lmb.cam.ac.uk/SUPERFAMILY/hmm.html) [51] was used to predict cellulose-binding domains. Protein sequences were aligned using ClustalX [52] and the resulting alignments were used as input to generate a phylogenetic tree using MEGA2.1 [53]. Statistical confidence of the nodes of the tree are based on 10,000 bootstrap replicates.

### Transgenic plants

The full-length *SlCOBRA-like* cDNA was isolated by reverse transcription (RT)–PCR from tomato seedlings using PrimeSTAR HS DNA Polymerase (TaKaRa) and gene-specific primers (Additional file 2: Table S1). The cDNA was cloned into the modified binary vector pBI121 to generate an overexpression construct driven by the fruit-specific *TFM7* promoter (Accession no.X95261) [25]. Transgenic plants were generated by *Agrobacterium tumefaciens*-mediated transformation as described by Fillatti et al. [54]. Transformed lines were selected on medium containing kanamycin (70 mg/L) and further confirmed by PCR for the presence of the NPTII (Kan^r^) marker gene (Additional file 2: Table S1). After RT-PCR analysis to verify *SlCOBRA-like* mRNA accumulation in the positive transgenic lines, three independent overexpressing lines (TFM7-OE) and three independent co-suppression lines (TFM7-CS) were identified from the primary transgenic (T0) population.

### Gene expression analysis

Total RNAs were extracted using Trizol reagent following the protocol provided by the manufacturer (Invitrogen, Carlsbad, CA) and treated with DNase (TaKaRa, Dalian, China). About 1 μg of total RNA from each sample was used for first-strand cDNA synthesis. For real-time quantitative RT-PCR, the PCR reaction was performed using SyBR Green PCR Master Mix (Applied Biosystems) and gene-specific primers (Additional file 2: Table S1) on the iCycler PCR system (BIO-RAD, Hercules, California, USA). Each sample was amplified in triplicate. REST software [55] was used to quantify the mRNA levels of *SlCOBRA-like* and other selected genes with the *UBI3* gene (Accession no.X58253) serving as the internal reference. Normalization was performed by the 2-Ct method. All primers used in this work are listed in Additional file 2: Table S1.

### Histochemical staining and cytology

For fruit epidermis analysis, tissue was carefully isolated from the fruit surface with a razor blade, and the tissue-bound slide was rinsed twice in distilled water and mounted in 15% HCl under a cover slip and photographed using a Leica LDM 2500 microscope. Six exocarp slices from three different fruits per plant were isolated from identical positions of fruits at 15 DPA. For each exocarp slice, epidermal cell size (not including cell wall) and cell separation (spacing distance between cells) were measured at three different positions (10 cells each position) using the ImageProPlus software (IPP6.0, Media Cybernetics, Inc.). Values represent the means of 180 (6×30) cells.

For cytological assesment, three fruits per plant were collected at the MG stage. Fresh hand-cut pericarp sections (~0.1 mm thick) were incubated in a 0.005% aqueous solution of calcofluor (fluorescent brightener 28; Sigma) for 2 min [18] and visualized with a fluorescent microscope (Leica, Wetzlar, Germany). To examine pericarp cell wall structure, paraffin-embedded transverse sections (10 μm in thickness) were obtained using a Leica microtome (RM 2265, Meyer Instruments, Inc) and stained with safranin O (a cell wall-specific dye), followed by photography using a Leica microscope (LDM 2500).

### Cell wall material (CWM) isolation and cell wall component analysis of RR fruit pericarp

Three RR fruits per plant were collected from non-transformed WT and three independent overexpression or co-suppression lines, respectively, and then their pericarp tissues were mixed, rapidly ground into fine powder in liquid nitrogen, and stored at −80°C until use. In each case, approximately 15 g frozen power was incubated with 70% ethanol for 90 min at 70°C to prevent autolytic activity. Insoluble material was washed sequentially with 95% ethanol, chloroform:methanol (1:1, v/v), and acetone. The dried pellets constituted crude cell wall extract/alcohol insoluble solids [AIRs] and were assayed for cellulose content using anthrone as a coloring agent using α-Cellulose (Sigma) as the standard, according to methods described previously [30].

The remaining AIRs were subsequently extracted with 90% (v/v) dimethyl-sulfoxide (DMSO) for 22 h at room temperature to solubilize starch, ending with two washes of the wall pellets with acetone and dessication in a vacuum oven [31]. The pellets (the cell wall materials, CWM) were stored in a glass desiccator until use. To determine non-cellulosic sugar composition, about 5 mg CWMs was hydrolyzed with 2 M trifluoroacetic acid (TFA) containing 4 mM of myoinositol as an internal standard at 105°C for 3 h, and then the TFA-soluble fraction was converted to alditol acetates and analyzed by gas chromatography as described previously [32]. Equimolar standards were also converted to alditol acetates to calculate response factors for quantitation of mol% relative to the myoinositol standard.

Pectin fractionation was carried out following the procedure of Rose et al. (1998) [31]. About 100 mg CWMs was extracted with water, 50 mM CDTA in 50 mM sodium acetate (pH 6.0), 100 mM Na_2_CO_3_ containing 0.1% NaBH_4_, sequentially. The uronic acid (UA) content in the different pectin fractions was estimated colorimetrically using galacturonic acid as a calibration standard [56].

### FTIR spectroscopy

For FTIR spectra analysis, 6 RR fruits per plant from three independent overexpression, co-suppression or WT lines were collected, and the corresponding AIRs of pericarps from 54 (6×9) fruits were extracted as described above. In each case, AIR was spread thinly onto a barium fluoride window, dried on the window at 37°C for 20 min. An area of 50×50 μm was selected for analysis by FTIR microspectroscopy [57]. All data sets were baseline-corrected and area-normalized before statistical analyses were applied. Exploratory PCA was carried out using PASW statistics software 18 (formerly known as SPSS Statistics, SPSS Inc.). Reference IR absorption spectra of cellulose were used for peak assignments [27,58].

### Textural and shelf-life analysis

Fruit firmness was determined based on compression mass and skin puncture strength of fresh intact fruits collected at MG (35 DPA) and RR (7 days after Break), using TA-XT Plus (Stable Microsystems Texture Analyser, UK). For the compression test, 15 fruits per plant were assayed at each stage. Each fruit was compressed to a 50% strain at the test speed of 2 mm s^−1^ with a 100 mm compression platen (P/100) and 10 g of applied force. Skin puncture strength and penetration distance of fresh intact fruits were measured by penetration using a 2mm Cylinder Probe (P/2N) with a trigger force of 5 g, loading at 2 mm s^-1^ to reach a 50% strain. Each fruit was tested three times at equidistant points along the equatorial plane of the fruit. 6 fruits per plant were taken at each stage. Values represent means ±SE (n=18).

For shelf life, fruits at the RR stage were detached and kept at room temperature (23~25°C and 55~60% relative humidity) for approximately 40 days. 6 replicates were taken for each individual plant. Average fresh weight loss was determined every 5 days until they lost their texture and structural integrity.

### Statistical analysis

Statistical analysis was performed using PASW statistics software 18.0 (formerly known as SPSS Statistics, SPSS Inc.). For analyses of epidermal cells, cellulose content, pectin fractions, and sugar content, significance was calculated using the Student’s *t* test. For gene expression between WT and CS plants, a multiple comparison was performed by the LSD (Least significant difference) method.

### Accession number

Accession numbers for the *SlCOBRA-like* sequences reported in this article are BT013422 and JN398667. Other *SlCOBL* sequences were listed in Table 1. Sequence data in Figure 7 were listed in Additional file 2: Table S1. Other sequence data from this article can be found in GenBank under the following accession numbers:

Arabidopsis *At*COB(At5g60920), *At*COBL1 (At3g02210), *At*COBL2 (At3g29810), *At*COBL4 (At5g15630), *At*COBL5 (At5g60950), *At*COBL6 (At1g09790), *At*COBL7 (At4g16120), *At*COBL8 (At3g16860), *At*COBL9 (At5g49270), *At*COBL10 (At3g20580), *At*COBL11 (At4g27110); *Zea mays Zm*BK2 (ACF79122.1), *Zm*BK2L3 (NP_001104946), *Zm*BK2L6 (NP_001105970), *Zm*BK2L7 (NP_001105971.1), *Zm*COBL4 (EU955798.1); *Oryza sativa Os*BC1 (Os03g0416200), *Os*COBL2 (Os03g0416300), *Os*COBL3 (Os05g0386800), *Os*BC1L6 (Os07g0604300); *Os*COBL6 (Os07g0604400).

## Authors' contributions

YL, FX and JG conceived the study, designed the experiments and drafted the manuscript. YC and XT conducted the experiments. All authors read and approved the final manuscript.

## Supplementary Material

Additional file 1 **Figure S1.** Phylogenetic tree of COBRA-like homologs. Phylogenetic tree generated from the alignment of *Sl*COBL and other plant COBRA proteins. Scale bar represents the genetic distance and node numbers indicate bootstrap support values. Click here for file

Additional file 2 **Table S1.** Information about primers used in this work. Click here for file

Additional file 3 **Figure S2.** Sequence alignment of tomato COBRA proteins with AtCOB and OsBC1. The alignment was generated by ClustalX [50]. Gray and black shading indicated conservative changes and identical residues, respectively. Underlined residues corresponded to the HMM-predicted putative cellulose binding domain II. For *Sl*COBRA-like sequence, the Cys-rich highly conserved CCVS domain was indicated by asterisks, and conserved consensus N-glycosylation sites were indicated by black triangles. Click here for file
