# Supplementary figures and images for: Functional characterization of a tomato COBRA-like gene functioning in fruit development and ripening
Source: BMC Plant Biol. 2012 Nov 10;12:211. doi: 10.1186/1471-2229-12-211 (PMC3533923; doi:10.1186/1471-2229-12-211)

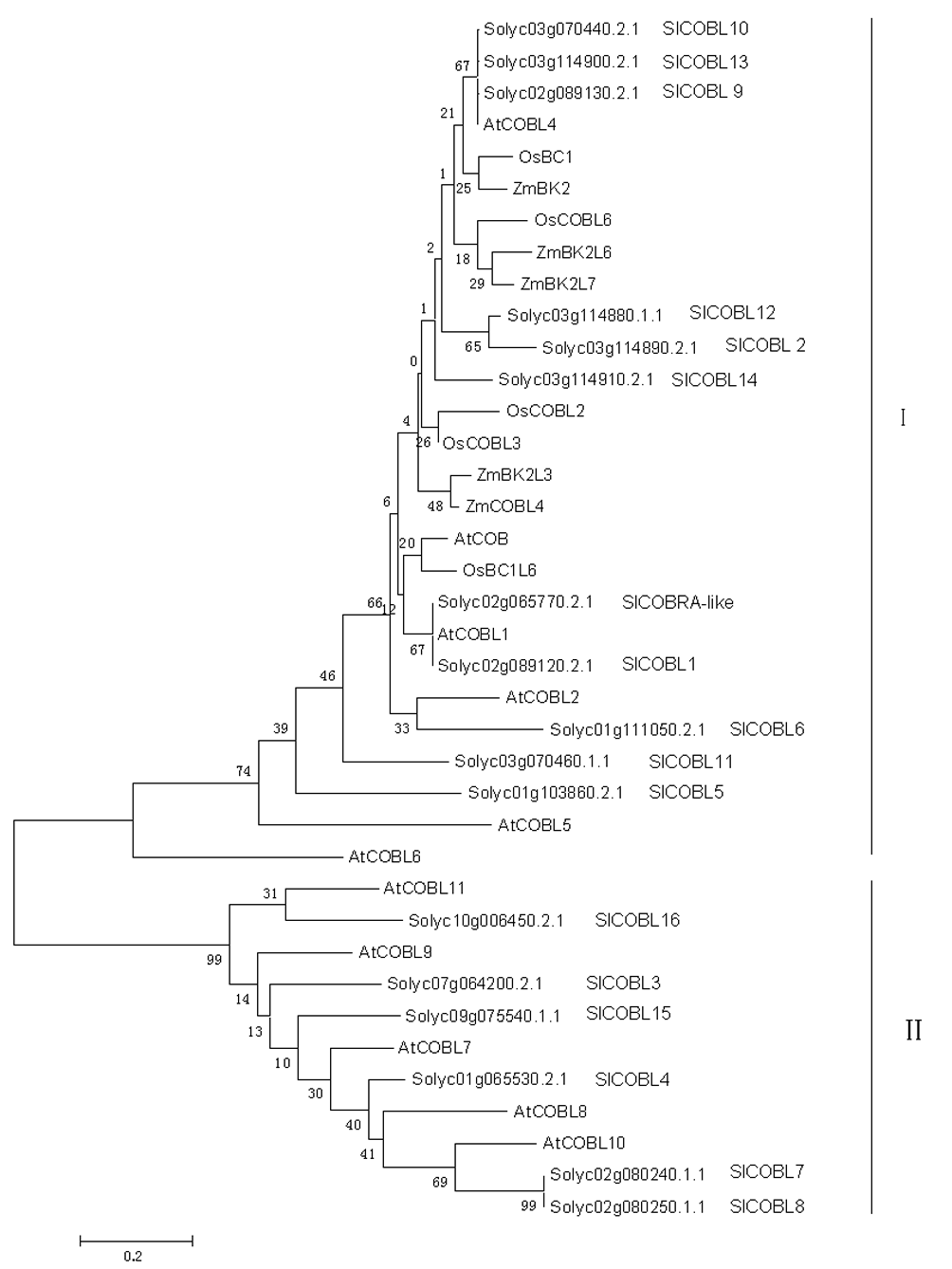

Supplement: Additional file 1 — Figure S1. Phylogenetic tree of COBRA-like homologs. Phylogenetic tree generated from the alignment of SlCOBL and other plant COBRA proteins. Scale bar represents the genetic distance and node numbers indicate bootstrap support values. [file 1471-2229-12-211-S1.tiff]

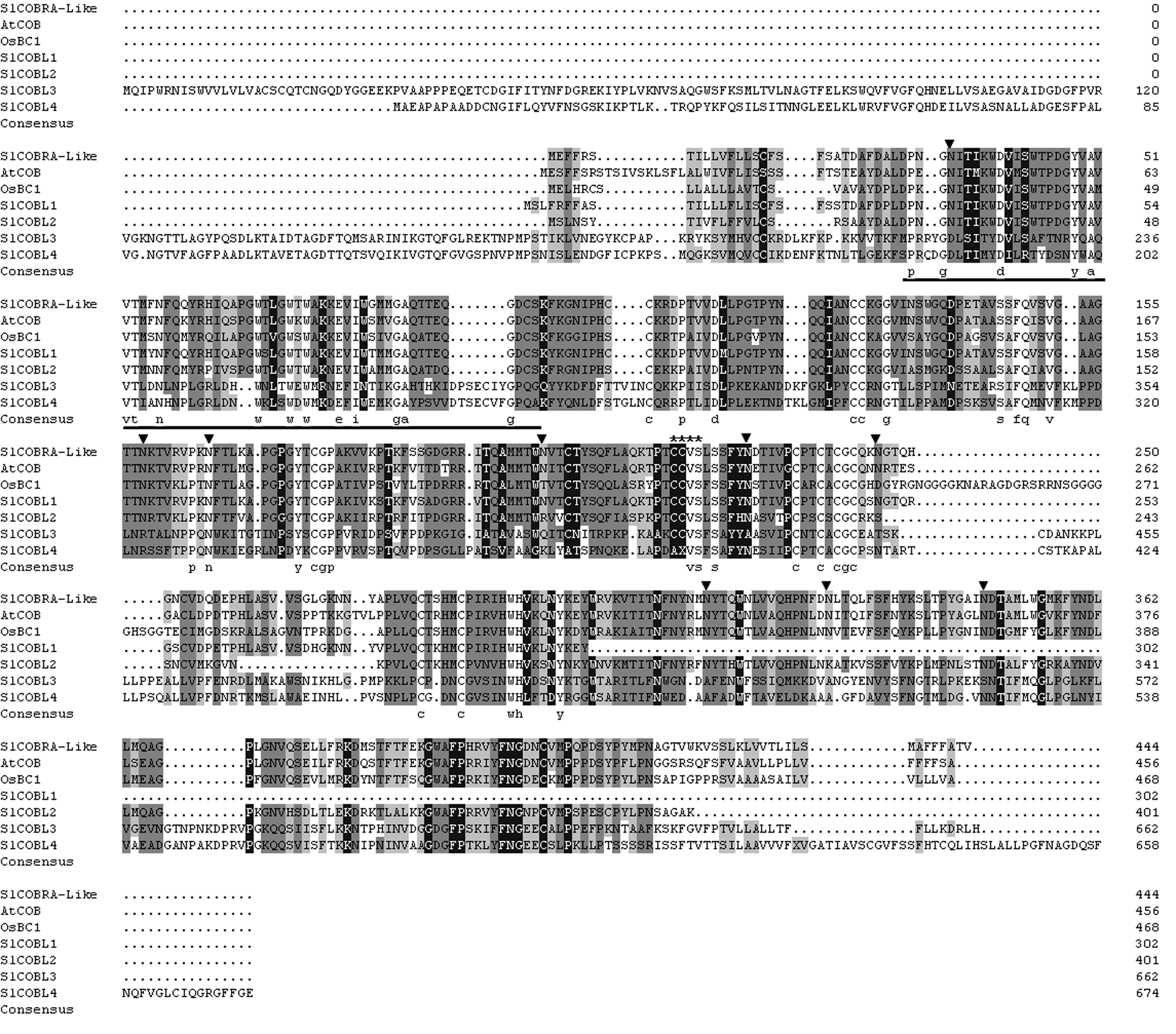

Supplement: Additional file 3 — Figure S2. Sequence alignment of tomato COBRA proteins with AtCOB and OsBC1. The alignment was generated by ClustalX [50]. Gray and black shading indicated conservative changes and identical residues, respectively. Underlined residues corresponded to the HMM-predicted putative cellulose binding domain II. For SlCOBRA-like sequence, the Cys-rich highly conserved CCVS domain was indicated by asterisks, and conserved consensus N-glycosylation sites were indicated by black triangles. [file 1471-2229-12-211-S3.tiff]
