# Supplementary material for: Functional characterization of a tomato COBRA-like gene functioning in fruit development and ripening
Source: BMC Plant Biol. 2012 Nov 10;12:211. doi: 10.1186/1471-2229-12-211 (PMC3533923; doi:10.1186/1471-2229-12-211)
Supplement: Additional file 2 — Table S1. Information about primers used in this work. [file 1471-2229-12-211-S2.doc]

## Additional file 3: Table S1 Information about primers used in this work

| Primer | Primer sequence (5'-3') | Target |
| --- | --- | --- |
| SlCOB-F(*Sma*I) | *CCCGGG*TGAACAGACATTGCCTACAGAGA | SlCOBRA-like overexpression |
| SlCOB- R(*Sac*I) | *GAGCTC*CAAGGTGTTTATCCGGTTTCT |  |
| Kan | GGCAATTACCTTATCCGCAA/ | NPTII (Kan^r^) |
|  | AGAACTCGTCAAGAAGGCGA |  |
| UBI3 real-F/R | AGAAGAAGACCTACACCAAGCC/ | UBI3 (X58253) |
|  | TCCCAAGGGT- TGTCACATACATC |  |
| SlCOB real-F/R | GGAGGAATTGGAGGGGACATTA/ | Tomato SlCOBRA-like (JN398667) |
|  | GAAATCATGGACTTTTCAGCATCA |  |
| WAK real-F/R | GGTAAGGGTCGTGTGGATGAGA/ | Tomato WAK-like kinase ( AY535423) |
|  | GCAAGGCATCTAAAAGCAAGC |  |
| THE1 real-F/R | CCTCTCTCCGAGCAAGGTGTTC/ | TC239291, tomato mRNA homologous with Arabidopsis THESEUS1 (NM_124818) |
|  | CACAGTTCTCTTCACAATAACCAATCA |  |
| LecRK real-F/R | CGGAGACGAAGCACATGACAA/ | TC227691,tomato mRNA homologous with Arabidopsis LecRK ( NM_115832) |
|  | AGTCCCTTGTAAACGGGTCCA |  |
| LeAGP1 real-F/R | GACAAGAACGCAAAGATAGGAA/ | Tomato arabinogalactan proteins (U81033.1) |
|  | AATGATTATCCCTGATCGTATGTAG |  |
| FEI real-F/R | CAATGTATTTGCCGTGAAGAGAA/ | TC232518, tomato mRNA homologous with Arabidopsis FEI1(NP_174427) |
|  | TCCTCGCAGATTTACCAGATACC |  |
| LeEXP1 real-F/R | TCGCCCTCACTTTGACCTCGCTA/ | Tomato expansin (U82123) |
|  | TCTGATTCCTCCTTGCTTTCGGC |  |
| TBG4 real-F/R | GATTGTCTTGGCAGTCATAC/ | Tomato β-galactosidase  (AF02039) |
|  | TACCAGCTCTTAACTTCACG |  |
| TBG6 real-F/R | CAAGAACGCAATGAAAGGGTAT/ | Putative tomato β-galactosidase (AF154424) |
|  | CGAGAACCATCCACTCCAAGC |  |
| PG real-F/R | TGGGATACTCCTAGCCGTAC/ | Tomato polygalacturonase (AF118567) |
|  | ACCACCCTGCCATGTCTTA |  |
| PME real-F/R | TAAATGGAAAGGGCACAAG/ | Tomato pectin methylesterase (U49330) |
|  | TCCTCAGCACGAATAGAAGT |  |
| SlCOBL1 real-F/R | TCAAGTTAGTGTTGGTGCTGCTG/ | Solyc02g089120.2.1 |
|  | GACATTCCAAGTCATCATAGCCTG |  |
| SlCOBL2 real-F/R | GTGGTCCTGCTAAAATCATCCG/ | Solyc03g114890.2.1 |
|  | ACTCTCCATGTCATCATAGCCTGTG |  |
| SlCOBL4 real-F/R | ATAACTAACAACGGACTTGAGGAAC/ | Solyc01g065530.2.1 |
|  | TACCATTACCAACTAAAGCAGGAA |  |
